# Subclinical Cardiovascular Adaptation in Uncomplicated Monochorionic Twin Pregnancies: Echocardiographic and Cardiac Biomarker Findings

**DOI:** 10.3390/diagnostics16203370

**Published:** 2026-10-16

**Authors:** Marta Campillo Ajenjo, Beatriz Herrero Ruiz, Laura Sotillo Mallo, María Carmen Bravo Laguna, Olaia Rodríguez Fraga, Bárbara Moreno Sanz-Gadea, Rebeca Sánchez Salmador, Helena Méndez del Sol, Roberto Rodríguez González, José Luis Bartha Rasero, Eugenia Antolín Alvarado

**Affiliations:** 1 Department of Obstetrics and Gynecology, La Paz University Hospital, 28046 Madrid, Spain; 2 Department of Neonatology, La Paz University Hospital, 28046 Madrid, Spain; 3 Laboratory Medicine Department, La Paz University Hospital, 28046 Madrid, Spain

**Keywords:** uncomplicated monochorionic twin pregnancy, fetal cardiac function, neonatal echocardiography, cardiovascular adaptation, NT-proBNP, cardiac troponin

## Abstract

**Background**: Although cardiac involvement is well characterized in complicated monochorionic (MC) pregnancies, whether subtle cardiovascular changes occur in uncomplicated pregnancies remains unclear. We characterized fetal and neonatal cardiovascular adaptation using echocardiography and cardiac biomarkers. **Methods**: In this prospective longitudinal cohort, 76 dichorionic (DC) and 68 uncomplicated monochorionic diamniotic (MCDA) twins underwent fetal echocardiography at 28 weeks and neonatal echocardiography within 72 h after birth. Cord blood N-terminal pro-B-type natriuretic peptide (NT-proBNP), high-sensitivity cardiac troponin I (hs-cTnI) and heart-type fatty acid-binding protein (H-FABP) were assessed at delivery. Analyses used generalized estimating equations to account for within-pregnancy clustering and Benjamini–Hochberg correction for multiple comparisons. Associations between cardiac biomarkers and fetal echocardiographic parameters and interactions with chorionicity and gestational age at birth were also evaluated. **Results**: MCDA fetuses showed trends toward a higher cardiothoracic ratio, lower right ventricular fractional shortening and tricuspid annular plane systolic excursion, and a higher prevalence of tricuspid regurgitation (TR) compared with DC fetuses. In the early neonatal period, nominal differences in interventricular septal thickness and tissue Doppler indices did not persist after FDR correction. Cord-blood hs-cTnI (21.83 vs. 12.29 pg/mL; *p* = 0.015, FDR-adjusted *p* = 0.027) and NT-proBNP (1327.6 vs. 887.6 pg/mL; *p* = 0.018, FDR-adjusted *p* = 0.027) concentrations were higher in MCDA twins. After gestational age adjustment, the association with hs-cTnI remained relatively stable, whereas that with NT-proBNP was attenuated; neither remained significant after FDR correction (*p* = 0.116 for both). The TR-hs-cTnI association differed by chorionicity (*p* for interaction < 0.001, FDR-adjusted *p* < 0.004) and persisted after adjustment for gestational age at birth. **Conclusions**: Conventional echocardiographic measures of cardiac structure and function appear to be largely preserved in uncomplicated MCDA twins during fetal life and the early neonatal period. Higher cord-blood hs-cTnI and NT-proBNP may suggest subclinical myocardial stress associated with monochorionicity, although gestational age-adjusted findings warrant caution.

## 1. Introduction

Cardiac remodeling is an adaptive mechanism in response to an insult, involving changes in the heart’s shape and size, as well as in different parameters of cardiac function [1]. Currently, fetal echocardiography can be used to assess fetal cardiac function and provide insight into fetal cardiovascular adaptation to a specific pathological condition. In addition, growing evidence supports the use of cardiac biomarkers as complementary tools for detecting myocardial stress and dysfunction [2,3].

Several clinical conditions have been associated with fetal cardiac dysfunction, including fetal anemia, vascularized tumors, congenital portosystemic shunt and intrauterine growth restriction, among others [1,4,5]. In this context, monochorionic (MC) twin pregnancy represents a particularly relevant model, given the unique hemodynamic environment to which these fetuses are exposed. The shared placenta, characterized by inter-twin vascular anastomoses connecting both fetal circulations, creates a dynamic pattern of blood exchange that is inherently susceptible to disruption. When this balance is compromised, serious complications may arise, most notably twin-to-twin transfusion syndrome (TTTS). In affected pregnancies, the recipient twin frequently exhibits a spectrum of cardiac findings—including cardiomegaly, myocardial hypertrophy, tricuspid regurgitation, and right ventricular outflow tract obstruction—that have been extensively documented in the literature [6,7,8,9]. Moreover, biochemical evidence of cardiac stress has been described in recipient fetuses, with increased levels of endothelin-1, B-type natriuretic peptide and cardiac troponin T [10,11]. Collectively, these findings reflect the adaptive response of the fetal heart to the pressure and volume overload imposed by the inter-twin hemodynamic imbalance.

Beyond these clinical entities, there is growing interest in understanding whether transient hemodynamic imbalances in MC gestations—even in the absence of overt complications—may nonetheless give rise to subclinical alterations in fetal cardiac function. Emerging evidence suggests that, although standard echocardiographic parameters remain within normal reference ranges, fetuses from MC pregnancies exhibit signs of cardiac adaptation compared with singletons, including atrial dilatation and cardiac hypertrophy accompanied by increased cord blood B-type natriuretic peptide (BNP) concentrations at birth [12]. However, whether biochemical markers of myocardial stress in uncomplicated MC twins parallel—or extend beyond—echocardiographic evidence of cardiac adaptation remains unclear.

Despite its potential relevance to long-term cardiovascular risk, available data are still limited, particularly regarding postnatal outcomes. In this context, a more comprehensive characterization of cardiac function in this population could contribute to improving early risk identification and developing more precise monitoring and prevention strategies.

Therefore, the aim of the present study was to compare cardiac function between uncomplicated MC and dichorionic (DC) twin pregnancies through prenatal and early postnatal echocardiographic assessment, together with biochemical markers of myocardial stress measured at delivery. We further aimed to provide a multimodal characterization of cardiac adaptation in uncomplicated MC twins.

## 2. Materials and Methods

A prospective longitudinal cohort study was conducted, including twin-pregnant women evaluated in the Fetal Medicine Unit at Hospital Universitario La Paz (Madrid, Spain) between 2020 and 2024. Based on chorionicity, two study groups were defined: uncomplicated DC twin gestations and uncomplicated monochorionic diamniotic (MCDA) twin gestations. No prospective sample-size calculation was performed; instead, all consecutive eligible pregnancies during the predefined study period were enrolled to maximize the available sample and minimize selection bias.

Inclusion criteria comprised uncomplicated twin pregnancies, maternal age of 18 years or older, and provision of written informed consent. Exclusion criteria included maternal age under 18 years, known fetal congenital defect, and complicated twin pregnancies of either DC or MCDA type. Uncomplicated twin pregnancies were defined by the absence of selective fetal growth restriction or intrauterine demise of one twin and, in MCDA pregnancies, by the additional absence of monochorionicity-specific complications, including twin-to-twin transfusion syndrome (TTTS), twin anemia–polycythemia sequence, and twin reversed arterial perfusion sequence. Maternal complications were not considered exclusion criteria.

Ethical approval was obtained from the institutional review board (approval HULP code: PI-3934).

Prenatal data were collected, including maternal age, type of conception, and type of pregnancy according to chorionicity. Data regarding the time of delivery were also collected, including mode of delivery, gestational age (GA) at delivery, birth weight and postnatal age at neonatal echocardiography expressed in hours.

A fetal ultrasound was performed at 28 weeks of gestation as part of the routine follow-up for both MCDA and DC twin pregnancies. Voluson E10 equipped with a convex and a 4D convex transabdominal multifrequency transducers were used (GE Medical Systems, Zipf, Austria). A routine ultrasound was performed on each twin to determine estimated fetal weight (EFW), deepest vertical pocket (DVP) of amniotic fluid, and Doppler study (umbilical artery (UA) pulsatility index (PI), middle cerebral artery (MCA) PI, ductus venosus (DV), peak systolic velocity in the MCA (MCA-PSV), as well as umbilical vein diameter and peak velocity).

The following cardiac functional parameters were determined by echocardiography. Two-dimensional (B-mode) echocardiography was used to obtain the cardiothoracic ratio (CTR), aortic and pulmonary diameters (mm), expressed as absolute values and normalized by EFW, and the presence or absence of tricuspid regurgitation (TR). M-mode echocardiography was used to measure left and right ventricular end-diastolic and end-systolic diameters (LVEDD, LVESD, RVEDD, and RVESD, mm), left and right ventricular free-wall thickness and interventricular septal thickness at end-diastole (LVFWd, RVFWd, and IVSd, mm), as well as mitral and tricuspid annular plane systolic excursion (MAPSE and TAPSE, respectively). Left and right ventricular fractional shortening (LVFS and RVFS) were calculated as LVFS (%) = [(LVEDD − LVESD)/LVEDD] × 100 and RVFS (%) = [(RVEDD − RVESD)/RVEDD] × 100, respectively. Pulsed-wave Doppler was used to assess mitral and tricuspid valve inflow, including peak early (E-wave) and late (A-wave) diastolic velocities and the corresponding E/A ratios. Peak systolic velocities and velocity–time integrals (VTIs) were obtained at the aortic and pulmonary outflow tracts. Left and right ventricular stroke volumes (LVSV and RVSV, respectively) were calculated from the corresponding semilunar valve diameter and VTI according to the formula SV = π × (D/2)^2^ × VTI, with aortic measurements used for LVSV and pulmonary measurements for RVSV; SV values were expressed both as absolute values and normalized by EFW. Isovolumetric contraction time (ICT), ejection time (ET), and isovolumetric relaxation time (IRT) were also assessed by pulsed-wave Doppler. The left ventricular myocardial performance index (LV MPI) was calculated as (ICT + IRT)/ET. The right ventricular myocardial performance index (RV MPI) was calculated as (a − b)/b, where *a* represents the interval from the end to the onset of tricuspid inflow and *b* represents the pulmonary ejection time. Tissue Doppler imaging (TDI) was used to assess myocardial velocities. TDI parameters included peak systolic (s′), early diastolic (e′), and late diastolic (a′) myocardial velocities at the mitral and tricuspid annuli.

Standard echocardiographic planes and measurements were acquired in accordance with the Spanish Society of Gynecology and Obstetrics (SEGO) guidelines [13]. Fetal echocardiographic examinations were performed by four experienced fetal medicine specialists as part of routine clinical follow-up; therefore, observers were not blinded to chorionicity.

At delivery, umbilical cord blood samples were collected from each twin. Blood samples were collected in serum tubes and processed in the laboratory. After clotting for 1 h, serum was separated by centrifugation at 3500 rpm for 10 min at room temperature. Aliquots were immediately stored at −80 °C until analysis. Biomarkers of myocardial injury (high-sensitivity cardiac troponin I [hs-cTnI] and heart-type fatty acid-binding protein [H-FABP]), myocardial dysfunction (N-terminal pro-B-type natriuretic peptide [NT-proBNP]) and inflammation (high-sensitivity C-reactive protein [hs-CRP]) were measured. hs-cTnI, NT-proBNP, and hs-CRP were quantified using chemiluminescence immunoassays on the Atellica Solution platform (Siemens Healthineers, Erlangen, Germany), with analytical ranges of 2.5–25,000 pg/mL, 35–35,000 pg/mL, and 0.16–10 mg/L, respectively. H-FABP was quantified using an enzyme-linked immunosorbent assay (ELISA; Hycult Biotech, Uden, The Netherlands) on a DSX system (DYNEX Technologies, Chantilly, VA, USA) with an analytical range of 0.1–10,000 pg/mL.

Finally, neonates underwent targeted functional neonatal echocardiography (TnEcho) within the first 72 h of life, including conventional echocardiography, pulsed-wave TDI [14], and Automatic Functional Imaging (AFI) [15]. TnEcho studies were performed with a Vivid S70 equipped with a sectorial multifrequency transducer (GE Medical Systems, Zipf, Austria) following the most recent European and American guidelines [16,17]. These parameters were adjusted for gestational age at birth and expressed as Z-scores [18,19,20,21,22,23]. Neonatal echocardiographic examinations were performed by four experienced neonatologists.

### Statistical Analysis

Statistical analysis was performed using SPSS version 29 for Windows (IBM Corp., Armonk, NY, USA). Continuous variables were assessed for normality using the Shapiro–Wilk test and visual inspection of histograms and Q-Q plots. Continuous variables were reported as mean ± standard deviation (SD) or median and interquartile range (IQR), according to their distribution, while categorical variables were reported as frequencies and percentages. Maternal and obstetric characteristics and perinatal outcomes defined at the pregnancy level were compared between DC and MCDA pregnancies using Student’s *t*-test or the Mann–Whitney *U* test for continuous variables and the χ^2^ test or Fisher’s exact test for categorical variables, as appropriate.

For analyses in which individual fetuses or neonates constituted the unit of analysis, generalized estimating equations (GEEs) were used to account for the non-independence of co-twins from the same pregnancy. Pregnancy was specified as the clustering variable, using an exchangeable working correlation structure and robust standard errors. Continuous outcomes were modeled using a Gaussian distribution with an identity link, whereas binary outcomes were analyzed using a binomial distribution with a logit link and are reported as odds ratios (ORs) with 95% confidence intervals (CIs).

Cardiac biomarker concentrations were natural-log transformed before analysis because of their positively skewed distributions. Differences in biomarker concentrations between DC and MCDA twins were assessed using GEE. Estimated marginal means and 95% CIs were back-transformed and are presented as geometric means on the original scale. Additional sensitivity analyses were performed by including gestational age at delivery as a continuous covariate in the GEE models to assess its potential influence on the association between chorionicity and biomarker concentrations. Both unadjusted and gestational age-adjusted estimates were reported.

Exploratory analyses were performed to investigate potential relationships between cardiac biomarkers and selected echocardiographic parameters (CTR, TAPSE, RVFS, and TR). These analyses were considered hypothesis-generating and were not intended to identify independent predictors of biomarker concentrations. Associations of hs-cTnI and NT-proBNP concentrations with each echocardiographic parameter were evaluated using separate GEE models accounting for within-pregnancy clustering. Potential effect modification by chorionicity was subsequently explored by including a chorionicity-by-echocardiographic parameter interaction term. Additional analyses including gestational age at birth were performed to assess its potential influence on the observed associations.

To account for multiple testing, the Benjamini–Hochberg procedure was used to control the false discovery rate (FDR). FDR correction was applied separately to clinically coherent families of prenatal echocardiographic variables, to neonatal echocardiographic parameters, and to cardiac biomarkers. For exploratory echocardiographic–biomarker associations and their interactions with chorionicity, correction was applied separately for each biomarker.

All tests were two-sided. Statistical significance was set at *p* < 0.05, or at FDR-adjusted *p* < 0.05 for analyses subject to multiple-testing correction.

## 3. Results

**Figure 1:**
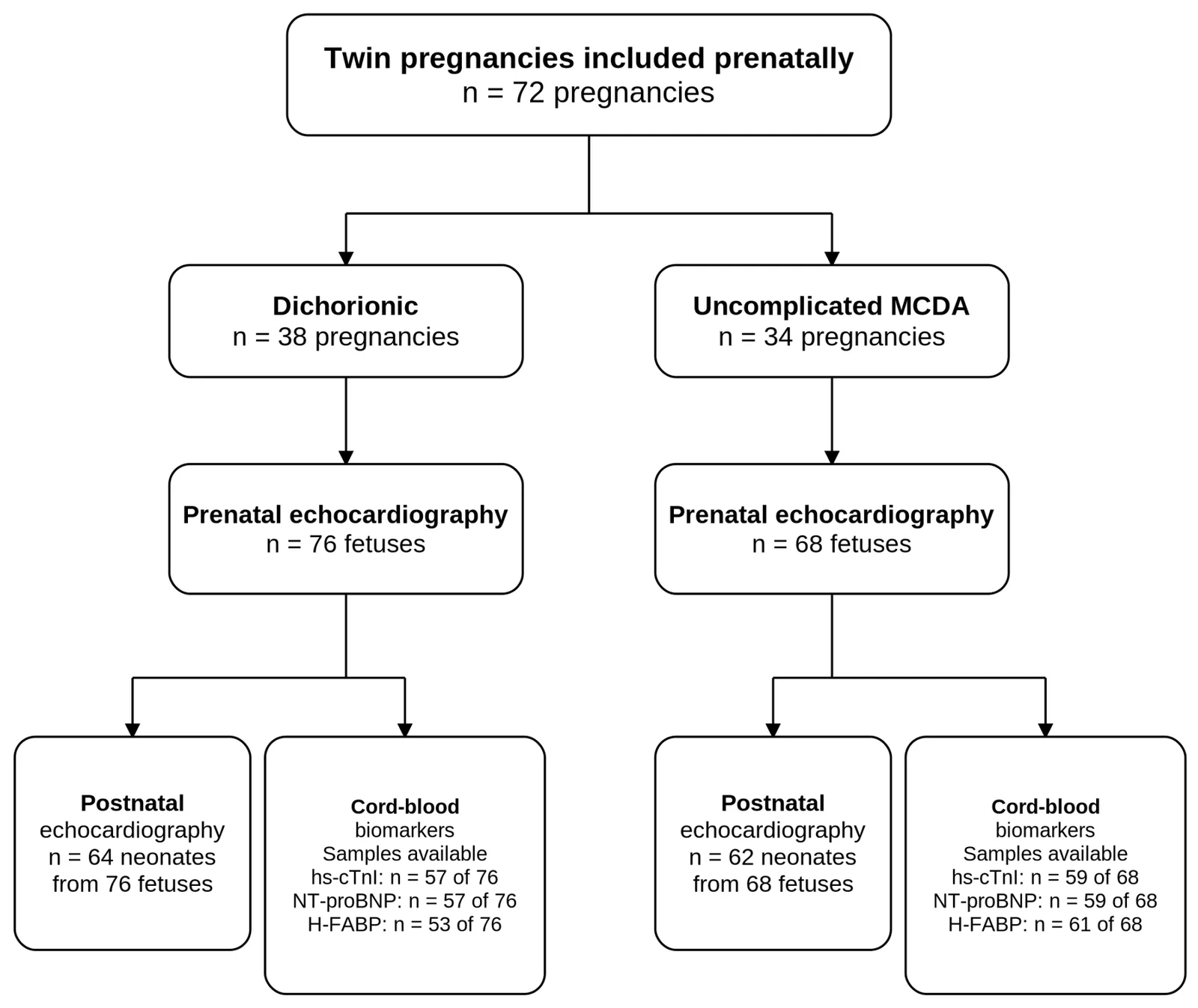
Study flowchart. Flow diagram of the study population from prenatal inclusion to postnatal follow-up, including fetal and neonatal echocardiography and umbilical cord blood sampling at delivery.

Figure 1 summarizes the study flow and the prenatal and postnatal assessments.

A total of 144 fetuses from uncomplicated twin pregnancies were included in the study, comprising 68 MCDA and 76 DC twins. Baseline maternal, pregnancy, and perinatal characteristics are presented in Table 1.

Fetal echocardiography showed a higher CTR in uncomplicated MCDA compared with DC twins (0.555 vs. 0.539; GEE *p* = 0.051), together with lower RVFS (31.91% vs. 35.59%; GEE *p* = 0.093) and TAPSE (6.56 vs. 6.91 mm; GEE *p* = 0.092). Tricuspid regurgitation was also more frequent in MCDA fetuses (19.1% vs. 9.2%; OR 2.33, 95% CI 0.77–7.03; GEE *p* = 0.134). None of these differences reached statistical significance. Absolute aortic and pulmonary artery diameters were lower in MCDA fetuses, although only the difference in pulmonary artery diameter remained significant after FDR correction. However, these differences were no longer observed after normalization by EFW. Similarly, RVSV was lower in absolute terms in MCDA fetuses, but no significant between-group difference was observed after normalization by EFW. Overall, no robust differences in fetal cardiac structure or function were observed after accounting for fetal size, within-pregnancy clustering and multiple testing (Table 2).

At the neonatal echocardiographic assessment, MCDA twins showed higher IVSd Z-scores than DC twins (estimated mean 0.834 vs. 0.212; mean difference 0.622, 95% CI 0.079–1.165; GEE *p* = 0.025), as well as higher septal e′/a′ (0.024 vs. −0.510; mean difference 0.534, 95% CI 0.069–0.998; GEE *p* = 0.024) and LV e′/a′ Z-scores (0.306 vs. −0.334; mean difference 0.640, 95% CI 0.007–1.273; GEE *p* = 0.048). However, none of these differences remained statistically significant after correction for testing (FDR-adjusted *p* = 0.118 for all three). No other neonatal echocardiographic parameter showed a significant between-group difference after FDR correction (Table 3).

Cord-blood hs-cTnI and NT-proBNP measurements were available for 116/144 fetuses (80.6%), and H-FABP for 114/144 (79.2%). Compared with pregnancies without biomarker data, those with available biomarker data had a higher gestational age at delivery (36.24 ± 1.40 vs. 34.79 ± 2.19 weeks; *p* = 0.007), with no significant differences in maternal age, chorionicity, mode of conception, or mode of delivery.

**Figure 2:**
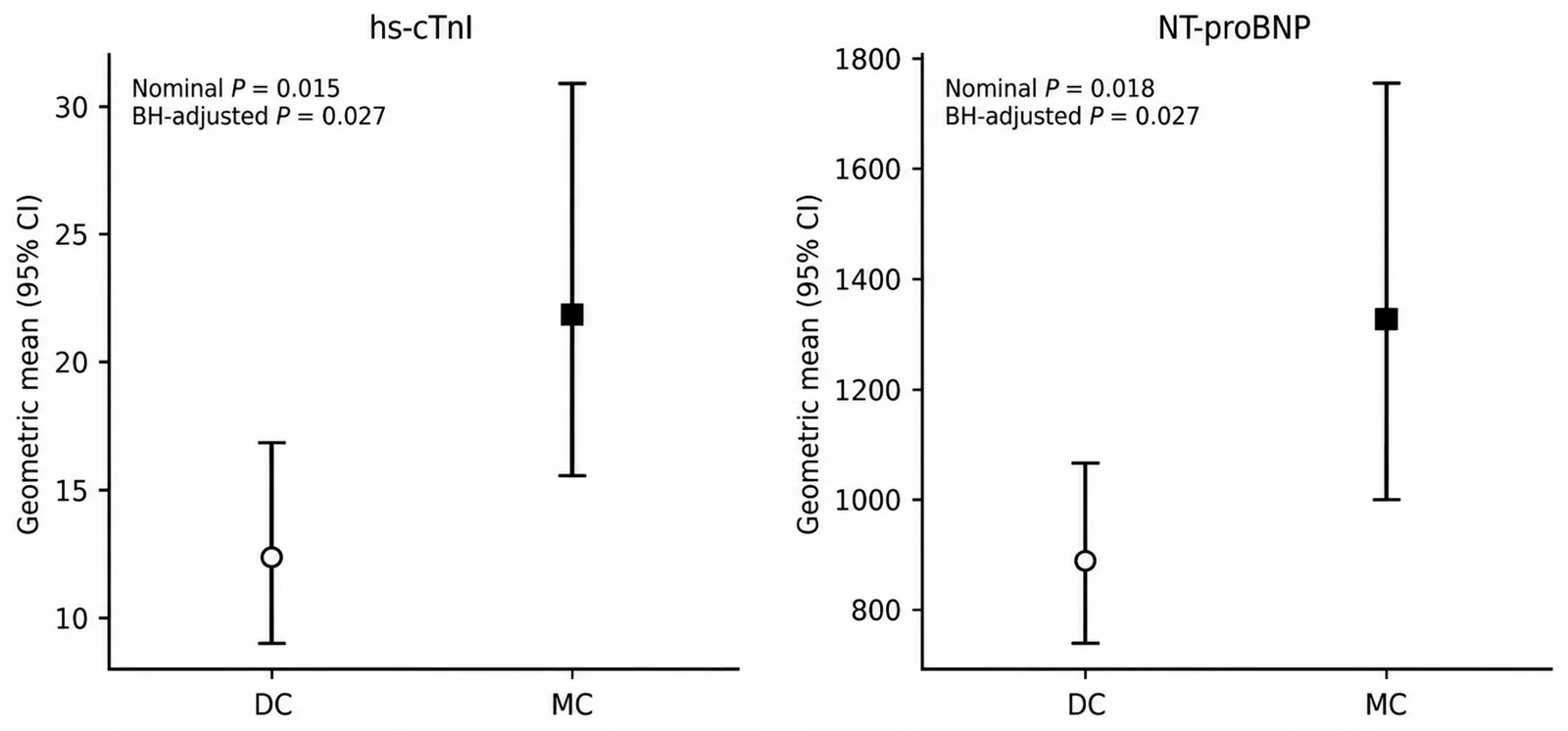
Cardiac biomarker concentrations according to chorionicity. Geometric mean concentrations and 95% confidence intervals (CI) of high-sensitivity cardiac troponin I (hs-cTnI) and N-terminal pro-B-type natriuretic peptide (NT-proBNP) in dichorionic (DC) and monochorionic (MC) twins. Estimates account for within-pregnancy clustering but are not adjusted for gestational age at delivery. *p* values were obtained from GEE models; FDR-adjusted *p* values were calculated using the Benjamini–Hochberg procedure.

Cardiac biomarker concentrations according to chorionicity are shown in Table 4. hs-cTnI concentrations were significantly higher in MCDA than in DC twins (geometric mean 21.83 vs. 12.29 pg/mL; geometric mean ratio MCDA/DC 1.78, 95% CI 1.12 to 2.82; *p* = 0.015) and remained significant after FDR correction (FDR-adjusted *p* = 0.027). NT-proBNP concentrations were also significantly higher in MCDA twins (1327.6 vs. 887.6 pg/mL; geometric mean ratio 1.50, 95% CI 1.07 to 2.09; *p* = 0.018), with the difference remaining statistically significant after FDR correction (FDR-adjusted *p* = 0.027) (Figure 2). No between-group difference was observed for H-FABP (4.24 vs. 3.97 pg/mL; geometric mean ratio 1.07, 95% CI 0.76 to 1.50; *p* = 0.705; FDR-adjusted *p* = 0.705). hs-CRP concentrations were below the lower limit of quantification (<0.5 mg/L) in 115 of 118 samples (97.5%), precluding a meaningful statistical comparison between chorionicity groups. Therefore, no inferential analysis of hs-CRP was performed.

After adjustment for gestational age at delivery, the association between monochorionicity and hs-cTnI remained relatively stable in magnitude (geometric mean ratio 1.697, 95% CI 1.004–2.868; *p* = 0.048), although it was no longer statistically significant after FDR correction (adjusted *p* = 0.116). In contrast, the association with NT-proBNP was attenuated (geometric mean ratio 1.312, 95% CI 0.971–1.773; *p* = 0.077; FDR-adjusted *p* = 0.116). No significant association was observed for H-FABP after adjustment (Supplementary Table S1).

No significant overall associations were observed between hs-cTnI or NT-proBNP concentrations and CTR, TAPSE, RVFS, and TR in GEE analyses accounting for within-pregnancy clustering, and all remained non-significant after FDR correction (Table 5).

**Figure 3:**
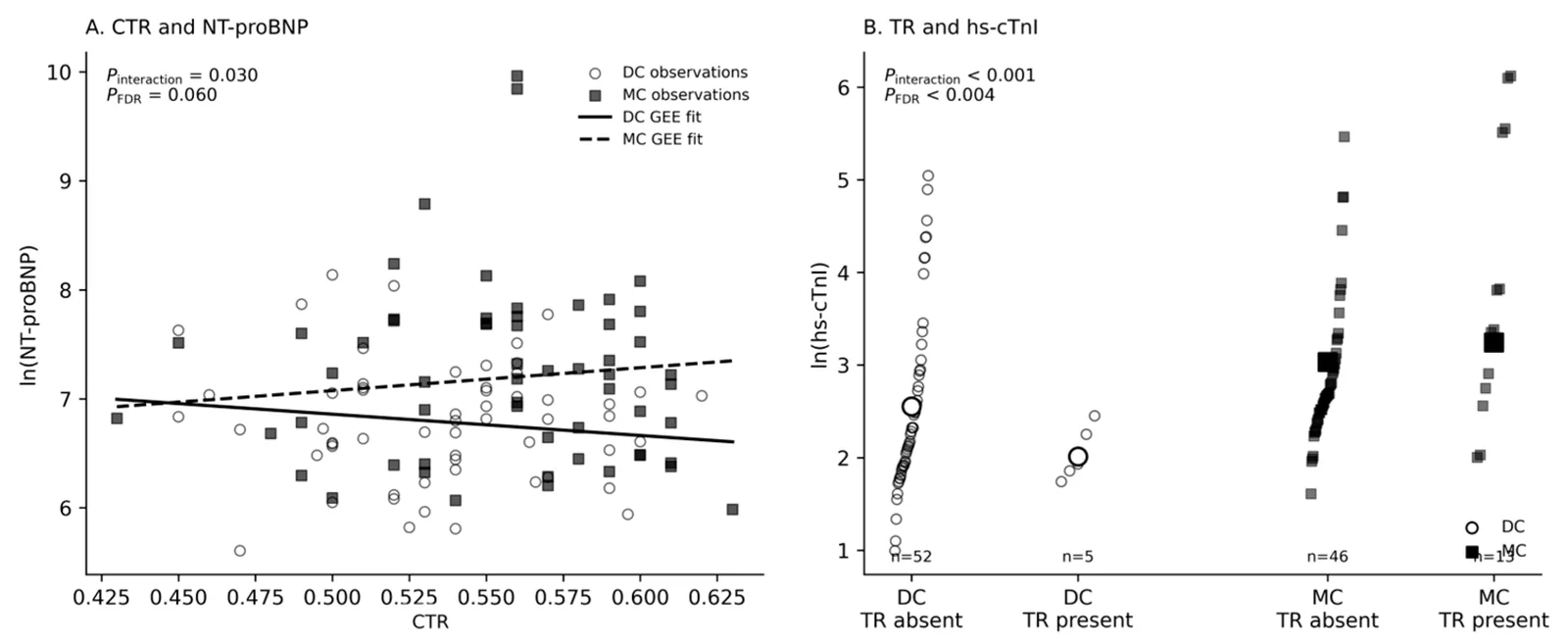
Effect modification by chorionicity in the association between echocardiographic parameters and cardiac biomarkers. (**A**) Association between cardiothoracic ratio (CTR) and NT-proBNP concentrations according to chorionicity. Solid and dashed lines represent GEE-estimated relationships for DC and MC twins, respectively. The interaction between CTR and chorionicity was nominally significant (*p* for interaction = 0.030) but did not remain significant after FDR correction (adjusted *p* = 0.060). (**B**) hs-cTnI concentrations according to the presence or absence of tricuspid regurgitation (TR), stratified by chorionicity. A significant interaction between TR and chorionicity was observed (*p* for interaction < 0.001; FDR-adjusted *p* < 0.004).

Potential effect modification by chorionicity was then evaluated. A significant interaction between chorionicity and TR was observed for hs-cTnI (*p* for interaction < 0.001), which remained significant after FDR correction (adjusted *p* < 0.004). Among MCDA twins, TR was associated with higher hs-cTnI concentrations, whereas this pattern was not observed in DC twins; however, this finding should be interpreted cautiously because only five DC fetuses with TR contributed to this comparison. For NT-proBNP, evidence of interaction was also observed for CTR and TR (*p* for interaction = 0.030 and 0.026, respectively), although neither remained significant after FDR correction (adjusted *p* = 0.060 for both). No evidence of effect modification by chorionicity was observed for TAPSE or RVFS (Supplementary Table S2; Figure 3).

Additional adjustment for gestational age at delivery affected the observed interactions differently for NT-proBNP and hs-cTnI. For NT-proBNP, the CTR-by-chorionicity interaction was attenuated (β = −3.787, 95% CI −7.589 to 0.015; *p* = 0.051), while the TR-by-chorionicity interaction remained significant before FDR correction (*p* = 0.025) but was no longer statistically significant after FDR correction (adjusted *p* = 0.100). In contrast, the TR-by-chorionicity interaction for hs-cTnI remained essentially unchanged after adjustment for gestational age (β = −0.736, 95% CI −1.147 to −0.325; *p* < 0.001) and remained significant after FDR correction (adjusted *p* < 0.004).

## 4. Discussion

In this prospective study, uncomplicated MCDA twins showed higher cord-blood hs-cTnI and NT-proBNP concentrations than DC twins, whereas conventional fetal and early neonatal echocardiographic parameters showed no robust differences between groups after accounting for within-pregnancy clustering and multiple testing. These findings suggest that biochemical evidence of myocardial stress may be detectable in uncomplicated MCDA twins even in the absence of clear echocardiographic abnormalities.

Prenatally, MCDA fetuses showed a tendency towards a higher CTR compared with DC fetuses, although this difference did not reach statistical significance. Previous studies comparing uncomplicated MCDA fetuses with singletons have similarly reported no differences in overall relative cardiac size, despite describing differences in cardiac morphology, including atrial dilation and smaller, more spherical ventricles with concentric hypertrophy [12]. Direct comparison with our findings should, however, be made with caution, as these studies used singleton pregnancies as the control group, whereas our study compared MCDA with DC twin pregnancies.

A similar non-significant pattern was observed in right ventricular systolic functional parameters, with lower RVFS and TAPSE in MCDA fetuses. Previous findings regarding systolic function in uncomplicated MCDA twins have not been entirely consistent. Earlier studies reported preserved or enhanced systolic function compared with singletons, as evidenced by increased longitudinal myocardial motion, higher ejection fraction, and higher cardiac output [12,24]. In contrast, a recent study using Fetal HQ reported lower indices of ventricular systolic function in uncomplicated MCDA fetuses, including lower LV ejection fraction, RV global longitudinal strain, fractional area change, and segmental fractional shortening [25]. Differences in the gestational age at assessment, echocardiographic techniques, and particularly the choice of singleton rather than DC twin controls may contribute to these apparently discrepant findings. In our cohort, the direction of the differences in RVFS and TAPSE may suggest subtle differences in right ventricular systolic function associated with monochorionicity. TR was also more frequent in MCDA fetuses, although this difference was not statistically significant. Overall, the observed differences in RVFS, TAPSE, and TR may suggest subtle right ventricular adaptation associated with monochorionicity; however, their lack of statistical significance warrants cautious interpretation.

During the early postnatal period, MCDA twins showed higher IVSd and septal and LV e′/a′ Z-scores compared with DC twins, although none of these differences remained statistically significant after correction for multiple testing. No significant differences were observed in longitudinal systolic function or cardiac output. These findings should be interpreted in the context of the substantial hemodynamic changes occurring during the fetal-to-neonatal transition. With the first breaths, lung aeration produces a rapid decrease in pulmonary vascular resistance and a marked increase in pulmonary blood flow, while cord clamping removes the low-resistance placental circulation and increases systemic vascular resistance. Consequently, ventricular preload shifts from predominantly umbilical to pulmonary venous return, and the LV becomes the systemic ventricle as the fetal shunts progressively close and the parallel fetal circulation is transformed into the neonatal series circulation [26]. These rapid changes in preload, afterload, and ventricular filling may influence load-dependent echocardiographic measures during the first days of life. Studies in healthy neonates have demonstrated changes in conventional and TDI measures during this transitional period, including an increase in E/A after birth, whereas systolic indices may remain relatively unchanged [27]. Thus, observed higher myocardial thickness may reflect subtle differences in early postnatal cardiac adaptation, while the higher TDI-derived e′/a′ ratios may reflect differences in the physiological adaptation of ventricular relaxation and filling after birth, although their clinical significance remains uncertain. Moreover, interruption of the shared placental circulation after birth removes the potential hemodynamic influence of placental vascular anastomoses and may contribute to differences between the fetal and neonatal findings. To our knowledge, this study is among the first to characterize cardiac function during the first 72 h of life in otherwise uncomplicated MC twins, extending the assessment of cardiovascular adaptation beyond fetal life and providing novel insight into its early postnatal evolution.

Biomarker analysis provides additional evidence supporting the presence of myocardial involvement. In adulthood, plasma cardiac markers are used in conjunction with echocardiographic parameters to assess cardiac dysfunction. NT-proBNP is a marker of myocardial wall stress and increases in response to ventricular volume or pressure overload [28]. Cardiac troponin, including hs-cTnI, reflects myocardial injury and cardiomyocyte damage. H-FABP is an early marker of myocardial injury [29], whereas hs-CRP is a nonspecific marker of systemic inflammation [30]. In TTTS, increased cardiac biomarker concentrations have been consistently reported, particularly in recipient fetuses. Van Mieghem et al. reported increased BNP and cardiac troponin concentrations in the amniotic fluid (AF) of recipient fetuses with TTTS, with both biomarkers correlating with echocardiographic parameters [2]. These findings provided biochemical evidence of increased myocardial stress. Subsequent studies have also reported increased BNP levels in the AF [31] and umbilical cord blood at birth [12] in uncomplicated MC twins.

In contrast to the limited echocardiographic differences, in our cohort, cord-blood cardiac biomarkers showed a more consistent pattern according to chorionicity. hs-cTnI concentrations were approximately 1.8-fold higher and NT-proBNP concentrations 1.5-fold higher in MCDA than in DC twins, and both differences remained statistically significant after FDR correction. Additional adjustment for gestational age at delivery affected these associations differently. The association with hs-cTnI remained relatively stable in magnitude, whereas that with NT-proBNP was attenuated. However, neither association remained statistically significant after FDR correction in the adjusted models. Overall, these findings raise the possibility that the cardiovascular environment associated with a shared monochorionic placenta may result in subtle myocardial stress in MCDA twins even in the absence of complications, although these observations should be interpreted cautiously in light of the gestational age-adjusted analyses.

However, higher biomarker concentrations were not consistently associated with the conventional fetal echocardiographic parameters evaluated, suggesting that biochemical evidence of myocardial stress may not be fully captured by conventional echocardiographic assessment.

Exploratory interaction analyses suggested that the relationship between echocardiographic findings and cardiac biomarkers may differ according to chorionicity. The most robust finding involved TR and hs-cTnI. Among MCDA twins, the presence of TR was associated with higher hs-cTnI concentrations, whereas no such association was observed in DC twins, resulting in a significant chorionicity-by-TR interaction that remained significant after FDR correction. This finding raises the possibility that TR may be associated with greater myocardial stress in MCDA fetuses. However, whether this reflects a specific hemodynamic effect of monochorionicity cannot be established from the present data, particularly given the small number of DC fetuses with TR (n = 5). For NT-proBNP, interactions with chorionicity were observed for CTR and TR, but neither remained statistically significant after FDR correction, and no evidence of effect modification by chorionicity was observed for TAPSE or RVFS.

The associations involving NT-proBNP appeared more sensitive to adjustment for gestational age at delivery. In particular, the statistical evidence for the interaction between CTR and chorionicity weakened after adjustment, suggesting that gestational age may partly influence the observed relationships with NT-proBNP. This is consistent with the possibility that the earlier gestational age at delivery of MCDA twins may influence NT-proBNP concentrations. In contrast, the chorionicity-dependent association between TR and hs-cTnI was substantially more robust remaining significant after FDR correction and essentially unchanged after adjustment for gestational age. This suggests that the observed interaction is unlikely to be explained by differences in gestational age at birth, although the small number of DC fetuses with TR again warrants caution.

Overall, these findings indicate that the higher cardiac biomarker concentrations observed in MCDA twins were not accompanied by clear differences in conventional echocardiographic measures of cardiac structure or function. This apparent dissociation suggests that cardiac biomarkers may reflect subtle myocardial stress that is not readily apparent on conventional fetal echocardiography.

The distinct hemodynamic environment associated with monochorionicity may contribute to these findings, possibly through chronic, subclinical variations in inter-twin blood flow, although the underlying mechanisms cannot be established from the present data. Importantly, our results do not provide evidence of overt cardiac dysfunction or remodeling.

From a clinical perspective, the significance of these observations remains uncertain. Whether such subtle cardiovascular differences persist over time or have implications for later cardiovascular health requires further longitudinal investigation.

### Strengths and Limitations

The main strengths of this study include the prospective design, the integrated assessment—fetal and early neonatal functional echocardiography together with umbilical cord blood cardiac biomarkers—the focus on uncomplicated MC pregnancies (a relatively understudied population), and, importantly, the use of DC twins as the comparison group, which helps distinguish cardiovascular changes specifically associated with monochorionicity from those related to twin pregnancy itself.

Several limitations should be acknowledged. First, no formal sample size calculation was performed, and the relatively small sample may have limited the ability to detect modest between-group differences, particularly for infrequent findings and interaction analyses. Fetal echocardiographic assessment was performed at a single gestational time point, limiting the evaluation of changes in cardiac parameters throughout pregnancy. In addition, echocardiographic measurements were not performed blinded to chorionicity, and formal interobserver reproducibility was not assessed. Cord-blood biomarkers were not available for the entire cohort, which may have introduced some degree of selection bias. Finally, postnatal follow-up was limited to the first days of life, precluding assessment of longer-term cardiovascular outcomes.

## 5. Conclusions

In conclusion, uncomplicated MCDA twins showed an overall preserved cardiac phenotype from fetal life to the early neonatal period, with only subtle echocardiographic differences compared with DC twins. Higher cord-blood hs-cTnI and NT-proBNP concentrations suggest that monochorionicity may nevertheless be associated with subclinical myocardial stress, potentially related to its distinct hemodynamic environment, and not fully reflected by conventional echocardiographic assessment, although gestational age at delivery may partly contribute to these differences. The clinical significance and long-term cardiovascular implications of these findings remain to be determined.

## Figures and Tables

**Table 1:** Maternal and perinatal characteristics in DC and uncomplicated MCDA pregnancies.

Variable	DC Pregnancies (n = 38)	Uncomplicated MCDA Pregnancies (n = 34)	*p*
**Maternal data**			
Age (years)	34.5 (4.7)	33.8 (4.4)	0.445
Type of conception (%)			0.218
- Spontaneous	55.2	64.7	
- Ovulation induction/artificial insemination	2.6	0	
- In vitro fertilization	42.1	35.3	
**Perinatal data**			
Delivery (%)			1
- Cesarean section	57.9	58.8	
- Vaginal	42.1	41.2	
Birth weight (g)	2433.8 (354.4)	2265.0 (348.2)	0.009
GA at birth (weeks)	36.43 (1.35)	35.54 (1.55)	<0.001
Postnatal age at echocardiography (h)	30.62 (19.35)	25.34 (13.13)	0.107

Values are presented as mean (standard deviation) or percentage, as appropriate. DC, dichorionic diamniotic; MCDA, monochorionic diamniotic, GA, gestational age.

**Table 2:** Fetal ultrasound, Doppler, and cardiac parameters in DC and uncomplicated MCDA twins.

Variable	DC GEE-Estimated Mean	Uncomplicated MCDA GEE-Estimated Mean	MCDA-DC Difference (95% CI)	*p*	FDR-Adjusted *p*
	**Ultrasound data**				
DVP of amniotic fluid (cm)	5.71	5.38	−0.33 (−0.83 to 0.17)	0.196	0.196
EFW (g)	1250.46	1163.37	−87.09 (−139.61 to −34.57)	0.001	0.003
EFW percentile	38.29	32.82	−5.46 (−13.48 to 2.55)	0.182	0.196
	**Doppler**				
UA PI	1.10	1.11	0.012 (−0.053 to 0.076)	0.725	0.725
MCA PI	2.14	1.94	−0.198 (−0.382 to −0.015)	0.034	0.103
MCA-PSV (cm/s)	37.75	36.39	−1.37 (−4.14 to 1.40)	0.332	0.509
DV PI	0.56	0.48	−0.079 (−0.142 to −0.015)	0.015	0.090
Umbilical vein diameter (mm)	4.70	4.82	0.119 (−0.172 to 0.409)	0.424	0.509
Umbilical vein peak velocity (cm/s)	23.30	24.18	0.887 (−1.17 to 2.94)	0.398	0.509
	**Cardiac size and morphology**				
CTR	0.539	0.555	0.016 (−0.0001 to 0.033)	0.051	0.137
LVFWd (mm)	4.05	4.13	0.063 (−0.334 to 0.461)	0.754	0.973
RVFWd (mm)	3.96	3.90	−0.060 (−0.391 to 0.270)	0.720	0.973
IVSd (mm)	4.04	4.06	0.016 (−0.276 to 0.308)	0.913	0.973
Aortic diameter (mm)	4.60	4.37	−0.233 (−0.440 to −0.026)	0.027	0.110
Aortic diameter normalized by EFW	0.00372	0.00379	0.000071 (−0.000152 to 0.000293)	0.534	0.973
Pulmonary diameter (mm)	5.82	5.41	−0.403 (−0.671 to −0.134)	0.003	0.026
Pulmonary diameter normalized by EFW	0.00469	0.00469	0.000004 (−0.000249 to 0.000258)	0.973	0.973
	**Systolic function**				
RVFS (%)	35.59	31.91	−3.65 (−7.92 to 0.61)	0.093	0.249
LVFS (%)	44.19	42.24	−1.92 (−6.33 to 2.50)	0.395	0.527
MAPSE (mm)	5.03	4.99	−0.047 (−0.421 to 0.327)	0.807	0.893
TAPSE (mm)	6.91	6.56	−0.357 (−0.772 to 0.059)	0.092	0.249
RVSV (mL)	2.36	2.03	−33.20 (−60.06 to −6.34)	0.015	0.123
RVSV normalized by EFW	0.189	0.176	−0.0127 (−0.0332 to 0.0077)	0.223	0.356
LVSV (mL)	1.61	1.49	−11.96 (−28.13 to 4.22)	0.147	0.295
LVSV normalized by EFW	0.130	0.129	−0.0009 (−0.0143 to 0.0125)	0.893	0.893
	**Diastolic and global cardiac function**				
Mitral E/A ratio	0.778	0.742	−0.036 (−0.080 to 0.007)	0.104	0.648
Tricuspid E/A ratio	0.750	0.748	−0.002 (−0.038 to 0.033)	0.910	0.910
TR (%)	7/76 (9.2)	13/68 (19.1)	OR 2.33 (0.77–7.03)	0.134	0.648
ICT (ms)	33.34	34.99	1.64 (−1.34 to 4.63)	0.281	0.648
ET (ms)	168.25	170.01	1.76 (−3.49 to 7.01)	0.510	0.648
IRT (ms)	44.76	46.72	1.96 (−0.85 to 4.76)	0.172	0.648
LV MPI	0.470	0.486	0.015 (−0.021 to 0.052)	0.403	0.648
RV MPI	0.468	0.452	−0.016 (−0.073 to 0.041)	0.578	0.648
LV e′ (cm/s)	4.96	4.78	−0.172 (−0.545 to 0.201)	0.367	0.648
LV a′ (cm/s)	6.15	5.80	−0.339 (−0.942 to 0.264)	0.271	0.648
LV s′ (cm/s)	4.91	4.67	−0.221 (−0.688 to 0.247)	0.355	0.648
RV e′ (cm(s)	5.43	5.53	0.101 (−0.208 to 0.409)	0.523	0.648
RV a′ (cm/s)	7.62	7.38	−0.234 (−0.846 to 0.379)	0.455	0.648
RV s′ (cm/s)	5.39	5.29	−0.102 (−0.486 to 0.282)	0.602	0.648
	**Outflow parameters**				
Aortic Vmax (cm/s)	83.97	82.14	−1.82 (−6.12 to 2.47)	0.405	0.919
Aorta VTI (cm)	9.64	9.84	0.21 (−0.42 to 0.84)	0.518	0.919
Pulmonary Vmax (cm/s)	71.60	72.04	0.44 (−2.91 to 3.79)	0.797	0.919
Pulmonary VTI (cm)	8.69	8.71	0.03 (−0.51 to 0.56)	0.919	0.919

Data are presented as estimated marginal mean values unless otherwise indicated. Differences represent MCDA minus DC estimates. Continuous outcomes were analyzed using generalized estimating equations (GEE) with a Gaussian distribution and identity link, pregnancy as the clustering variable, an exchangeable working correlation structure, and robust standard errors. Tricuspid regurgitation was analyzed using binomial GEE with a logit link and is reported as odds ratio (OR) with 95% confidence interval. To account for multiple testing, the Benjamini–Hochberg procedure was applied within prespecified clinically coherent families of variables to control the false discovery rate (FDR). *p* values were obtained from generalized estimating equation (GEE) models accounting for within-pregnancy clustering; FDR-adjusted *p* values were calculated using the Benjamini–Hochberg procedure. DC, dichorionic diamniotic; MCDA, monochorionic diamniotic; DVP, deepest vertical pocket; EFW, estimated fetal weight; UA, umbilical artery; PI, pulsatility index; MCA, middle cerebral artery; MCA-PSV, middle cerebral artery peak systolic velocity; DV, ductus venosus; CTR, cardiothoracic ratio; LVFWd, left ventricular free wall thickness in diastole; RVFWd, right ventricular free wall thickness in diastole; IVSd, interventricular septal thickness in diastole; LV, left ventricle; RV, right ventricle; FS, fractional shortening; MAPSE, mitral annular plane systolic excursion; TAPSE, tricuspid annular plane systolic excursion; E/A, early-to-late diastolic filling velocity ratio; Vmax, maximum velocity; VTI, velocity-time integral; ICT, isovolumetric contraction time; ET, ejection time; IRT, isovolumetric relaxation time; MPI, myocardial performance index; SV, stroke volume; e′, early diastolic myocardial velocity; a′, late diastolic myocardial velocity; s′, systolic myocardial velocity.

**Table 3:** Neonatal echocardiographic parameters within the first 72 h of life in DC and uncomplicated MCDA twins.

Parameter (Z-Score)	DC GEE-Estimated Mean	Uncomplicated MCDA GEE-Estimated Mean	MCDA-DC Difference (95% CI)	*p*	FDR-Adjusted *p*
IVSd	0.212	0.834	0.622 (0.079 to 1.165)	0.025	0.118
IVSs	0.193	0.508	0.315 (−0.193 to 0.822)	0.224	0.320
TAPSE	−0.035	0.052	0.087 (−0.593 to 0.767)	0.802	0.802
MAPSE	−0.281	−0.078	0.203 (−0.552 to 0.957)	0.599	0.666
LVFS	−0.607	−0.315	0.292 (−0.011 to 0.595)	0.059	0.118
Right cardiac output	−0.486	−0.286	0.200 (−0.211 to 0.612)	0.341	0.426
Left cardiac output	−0.069	−0.685	−0.616 (−1.401 to 0.168)	0.124	0.207
Septal e′	0.419	0.935	0.516 (−0.009 to 1.040)	0.054	0.118
Septal e′/a′ ratio	−0.510	0.024	0.534 (0.069 to 0.998)	0.024	0.118
LV e′/a′ ratio	−0.334	0.306	0.640 (0.007 to 1.273)	0.048	0.118

Data are presented as GEE-estimated marginal means and are expressed as Z-scores. Differences represent MCDA minus DC estimates. *p* values were obtained from generalized estimating equation (GEE) models accounting for within-pregnancy clustering. FDR-adjusted *p* values were calculated using the Benjamini–Hochberg procedure. DC, dichorionic diamniotic; MCDA, monochorionic diamniotic; IVSd, interventricular septal thickness in diastole; IVSs, interventricular septal thickness in systole; FS, fractional shortening; LV, left ventricle; e′, early diastolic myocardial velocity; a′, late diastolic myocardial velocity; MAPSE, mitral annular plane systolic excursion; TAPSE, tricuspid annular plane systolic excursion.

**Table 4:** Umbilical cord biomarkers in DC and uncomplicated MCDA twins at birth.

Biomarker	DC Estimated Geometric Mean (95% CI)	Uncomplicated MCDA Estimated Geometric Mean (95% CI)	Estimated Ratio MCDA/DC (95% CI)	*p*	FDR-Adjusted *p*
hs-cTnI (pg/mL)	12.29 (9.03–16.73)	21.83 (15.45–30.84)	1.78 (1.12–2.82)	0.015	0.027
NT-proBNP (pg/mL)	887.6 (740.9–1063.3)	1327.6 (1003.4–1756.5)	1.50 (1.07–2.09)	0.018	0.027
H-FABP (pg/mL)	3.97 (3.25–4.84)	4.24 (3.20–5.63)	1.07 (0.76–1.50)	0.705	0.705

Values are GEE-estimated geometric means (95% CI) back-transformed to the original scale. Between-group effects are expressed as geometric mean ratios (MCDA/DC). *p* values were obtained from GEE models accounting for within-pregnancy clustering; FDR-adjusted *p* values were calculated using the Benjamini–Hochberg procedure. DC, dichorionic diamniotic; MCDA, monochorionic diamniotic; hs-cTnI, high-sensitivity cardiac troponin I; NT-proBNP, N-terminal pro-B-type natriuretic peptide; H-FABP, heart-type fatty acid-binding protein; GEE, generalized estimating equations; FDR, false discovery rate.

**Table 5:** Association between cardiac biomarkers and fetal echocardiographic parameters.

Echocardiographic Parameter	NT-proBNP β (95% CI)	*p*	FDR-Adjusted *p*	hs-cTnI β (95% CI)	*p*	FDR-Adjusted *p*
CTR	0.158 (−1.869 to 2.186)	0.878	0.961	0.644 (−2.421 to 3.709)	0.680	0.775
TAPSE	−0.021 (−0.075 to 0.034)	0.458	0.916	0.046 (−0.047 to 0.139)	0.334	0.775
RVFS	0.002 (−0.003 to 0.007)	0.360	0.916	0.002 (−0.005 to 0.010)	0.527	0.775
TR	0.008 (−0.310 to 0.326)	0.961	0.961	0.042 (−0.244 to 0.327)	0.775	0.775

Associations were assessed using generalized estimating equation (GEE) models accounting for within-pregnancy clustering. β coefficients represent the estimated change in natural-log-transformed biomarker concentration per unit increase in the echocardiographic parameter, for tricuspid regurgitation, β represents the estimated difference associated with its presence versus absence. *p* values were adjusted separately for each biomarker using the Benjamini–Hochberg false discovery rate (FDR) procedure. CTR, cardiothoracic ratio; TAPSE, tricuspid annular plane systolic excursion; RVFS, right ventricular fractional shortening; TR, tricuspid regurgitation; NT-proBNP, N-terminal pro-B-type natriuretic peptide; hs-cTnI, high-sensitivity cardiac troponin I; CI, confidence interval; FDR, false discovery rate.

## Data Availability

The data are not publicly available due to privacy and ethical restrictions.

## Supplementary Materials

The following supporting information can be downloaded at: https://www.mdpi.com/article/10.3390/diagnostics16203370/s1, Table S1: Association between chorionicity and cord-blood cardiac biomarkers before and after adjustment for gestational age at delivery. Table S2: Effect modification by chorionicity of the association between cardiac biomarkers and fetal echocardiographic parameters.
