# Supplementary material for: Subclinical Cardiovascular Adaptation in Uncomplicated Monochorionic Twin Pregnancies: Echocardiographic and Cardiac Biomarker Findings
Source: Diagnostics (Basel). 2026 Oct 16;16(20):3370. doi: 10.3390/diagnostics16203370 (PMC13703515; doi:10.3390/diagnostics16203370)
Supplement: Supplementary file 1 [file diagnostics-16-03370-s001.zip › diagnostics-4574382-supplementary.pdf]

Supplementary Table S1. Association between chorionicity and cord-blood cardiac biomarkers before and after adjustment for gestational age at delivery

**Biomarker**	**Model**	**Geometric mean ratio MCDA/DC (95% CI)**	***P***	***FDR-adjusted P***
hs-cTnI	Unadjusted for GA	1.775 (1.117–2.821)	0.015	0.027
	Adjusted for GA	1.697 (1.004–2.868)	0.048	0.116
NT-proBNP	Unadjusted for GA	1.496 (1.072–2.087)	0.018	0.027
	Adjusted for GA	1.312 (0.971–1.773)	0.077	0.116
H-FABP	Unadjusted for GA	1.068 (0.762–1.496)	0.705	0.705
	Adjusted for GA	0.956 (0.697-1.311)	0.781	0.781

Geometric mean ratios (MCDA/DC) and 95% CIs were estimated using GEE accounting for within-pregnancy clustering. Adjusted models included gestational age at delivery. FDR-adjusted P values were calculated using the Benjamini–Hochberg procedure separately for adjusted and unadjusted models. GA, gestational age at delivery; MCDA, monochorionic diamniotic; DC, dichorionic; hs-cTnI, high-sensitivity cardiac troponin I; NT-proBNP, N-terminal pro-B-type natriuretic peptide; H-FABP, heart-type fatty acid-binding protein; CI, confidence interval; FDR, false discovery rate.

Supplementary Table S2. Effect modification by chorionicity of the association between cardiac biomarkers and fetal echocardiographic parameters.

**Biomarker**	**Echocardiographic parameter**	**DC β**	**MCDA β**	**Difference in β (DC−MCDA)**	***p***	***FDR-adjusted P***
NT-proBNP	CTR	−1.950	+2.104	−4.054	0.030	0.060
	TAPSE	+0.015	−0.042	+0.057	0.343	0.343
	RVFS	+0.006	≈0.000	+0.006	0.220	0.293
	TR	−0.451	+0.169	−0.620	0.026	0.060
hs-cTnI	CTR	−0.032	+0.890	−0.922	0.761	0.841
	TAPSE	+0.037	+0.057	−0.020	0.841	0.841
	RVFS	+0.004	+0.001	+0.003	0.676	0.841
	TR	−0.541	+0.210	−0.751	<0.001	<0.004

β coefficients were estimated using generalized estimating equations (GEE), accounting for within-pregnancy clustering. Differences in β were calculated as DC − MCDA. P values correspond to the interaction between chorionicity and each echocardiographic parameter and were adjusted for multiple comparisons using the Benjamini–Hochberg false discovery rate (FDR) procedure, separately for each biomarker. Biomarker concentrations were natural-log transformed. DC, dichorionic; MCDA, monochorionic diamniotic; CTR, cardiothoracic ratio; TAPSE, tricuspid annular plane systolic excursion; RVFS, right ventricular fractional shortening; TR, tricuspid regurgitation; NT-proBNP, N-terminal pro-B-type natriuretic peptide; hs-cTnI, high-sensitivity cardiac troponin I; FDR, false discovery rate.
